# More Dental Schools

**Published:** 1879-09

**Authors:** 


### EDITORIAL, ETC.

---

*More Dental Schools.*—University Schools are multiplying. The Harvard and Ann Arbor and University of Pennsylvania Dental Schools are followed by the Dental Department of the Vanderbilt University, at Nashville, Tennessee ; and the prospectus of the Indiana Dental College, at Indianapolis, Indiana, at the same time appears. In the former, as the only familiar name, appears that of our time-honored old friend, Dr. W. H. Morgan, as Dean and Professor of Clinical Dentistry and Dental Pathology ; and in the former a familiar name is that of Dr. Joseph Richardson, the author of the "Mechanical Dentistry," of that name, as Professor of that branch.

If these new middle-western Schools will start out men who will crowd out and crowd down the unworthy who now so largely hold possession in those parts, as well as elsewhere, it will be well. But alas! who hopes for it? Rivalry is good indeed, up to a certain point, but let the dental profession take warning from the medical as to the danger and degradation attending unworthy and unlimited competition. When professors in a Medical School boldly hold the doctrine that the public will soon learn to discriminate in favor of the unworthy and that things soon, in spite of degrees, "adjust themselves," —in a word acknowledge that the degree in itself is worthless, though signed by them ; it is time to take the lesson to heart which these things teach ;—a plain lesson indeed, that there are two many medical schools.

We trust this is not to be construed as animadverting against these new schools. We do earnestly trust that they are the outgrowth of a "felt need" in the great West. If they develop into better schools than now exist, it will be the gain of dentistry. We publish their respective faculties in full, as follows:

*Indiana Dental College.*

P. G. C. Hunt,. M. D., D. D. S., Professor of Institutes of Dental Science.

John Chambers, M. D., C. M., Professor of Descriptive and Microscopic Anatomy.

Junius E. Cravens, D. D. S., Professor of Operative Dentistry, Dental Histology and Pathology, and Secretary.

William B. Fletcher, M. D., Professor of Physiology, Histo-Chemistry, and Pathology.

Milton H. Chappell, D. D. S., Professor of Clinical Dentistry.

C. E. Wright, M. D., Professor of Materia Medica and Therapeutics.

Joseph Richardson, D. D. S., Professor of Mechanical Dentistry and Metallurgy.

Henry Jameson, M. D., Professor of Chemistry and Diseases of Childhood Incident to Dentition.

*Dental Department of Vanderbilt University, Nashville, Tennessee.*

Dr. W. H. Morgan, Professor of Clinical Dentistry and Dental Pathology, and Dean.

Dr. J. C. Ross, Professor of Operative Dentistry and Dental Hygiene.

Dr. R. R. Freeman, Professor of Mechanical and Corrective Dentistry.

Dr. J. R. Buist, Professor of Oral Surgery and Surgical Pathology.

Dr. T. A. Atchinson, Professor of Materia Medica and Special Therapeutics.

Dr. D. R. Stubblefield, Professor of Anatomy and Physiology

Dr. R. W. Steger, Professor of Chemistry and Microscopy.

